# Urban-Rural Differences: The Impact of Social Support on the Use of Multiple Healthcare Services for Older People

**DOI:** 10.3389/fpubh.2022.851616

**Published:** 2022-04-14

**Authors:** Zhang Chi, Hu Han

**Affiliations:** School of Public Policy and Administration, Xi'an Jiaotong University, Xi'an, China

**Keywords:** urban-rural differences, older people, multiple healthcare services, informal social support, formal social support

## Abstract

**Background:**

There are many kinds of chronic diseases, high incidence and high hospitalization rate in older people caused by population aging. The increasing demand for healthcare services has become an increasingly prominent problem in Chinese society. The purpose of this paper is to explore the influence of social support on multiple healthcare services for older people and the urban-rural differences among them.

**Methods:**

The data are from our field survey in Shaanxi province in 2019. Using the Anderson model as the basic explanatory framework, this paper uses the Tobit-model to analyses the impact of social support for older people on the utilization of therapeutic healthcare services, and the Logit-model to analyze the impact of living arrangements and social support on the utilization of preventive healthcare services for older people.

**Results:**

This paper examines the impact of formal and informal support on outpatient, inpatient, and preventive healthcare services for older people, provides an in-depth analysis of the differences in the impact of social support on healthcare service utilization between urban and rural older people and analyses the contribution of various factors to the impact. The coefficient effect is divided into two parts: the coefficient “premium” of urban older people relative to urban and rural older people as a whole, which accounts for 10.8% of the total difference; and the “premium” of urban and rural older people as a whole to rural older people, which accounts for 18.9% of the total difference. The coefficient effect accounts for 29.7 per cent of the total difference.

**Conclusions:**

Rural older people place greater importance on the quantity of formal social support, while urban older people place greater importance on the quality of formal social support. The phenomenon of raising children for old age was evident in the use of healthcare services by rural older people, while the phenomenon of distant relatives being better than close neighbors was evident in the use of healthcare services by urban older people. Free preventive healthcare services in rural China have largely compensated for the lack of health benefits for rural older people.

## Background

The aging of the population has become an increasingly prominent issue in Chinese society (1). Along with the aging of the population and the increase in the number of older people, China has become the country with the largest number of older people in the world, and according to the data of the seventh census, there are 260 million people aged 60 and above in China, including 190 million people aged 65 and above, and the proportion of people aged 65 and above in China has reached 13.50% (2). In recent years, the aging of the population has led to significant changes in China's disease spectrum, with ~80% of older people suffering from at least one chronic disease (3). Older people are characterized by a wide range of chronic diseases, seriousness of illness, high morbidity rates and high hospitalization rates, and the demand for health care services is growing. Since 2012, China has gradually established a universal health insurance system and the level of protection has been increasing year on year, which, together with the increase in income, has boosted the consumption capacity of healthcare services for older people (4). As older people often need to be accompanied to health appointments, social support is an important factor influencing the use of healthcare services for older people. Basic healthcare utilization ensures that older people have the right to health, and equity in healthcare utilization for older people is a form of social justice. In recent years, the urban-rural divide in access to healthcare has become more pronounced.

The proportion of older people population in the country is 52% in urban areas and about 48% in rural areas. The proportion of the urban older people population is lower than the current urbanization rate of 63.89%. Due to the long-standing urban-rural dichotomy, there are significant differences in social support and access to healthcare services between rural and urban older people (5). As a result of family planning policies, many urban older people have only one child and are often away from their children, while rural older people generally have more than two children, and it is more common for rural older people to live with their children (6). Urban seniors are mostly better off than rural seniors in terms of income, access to healthcare and health insurance, urban seniors are better off than rural seniors in terms of average life expectancy and habitat, and rural seniors generally have a higher number of children than urban seniors (7). This shows that there is indeed a huge imbalance and even inequity in the social support received by the two. It is necessary to study the difference in the impact of health social support on healthcare services between urban and rural older people, in order to reduce the gap in access to healthcare services between urban and rural older people (8).

This paper expands traditional social support into formal support (government support for older people) and informal support (referring to an individual's emotional experience of understanding older people). The use of healthcare services is divided into diagnostic healthcare services and preventive healthcare services. Preventive healthcare services play an important role in the prevention of disease in older people, but this is a key factor that has rarely been considered in previous studies.

## Literature Review

### Social Support

The concept of social support as a scientific term was formally introduced in the psychiatric literature in the early 1970s, and as research on the concept continued in many fields such as sociology and psychology, social support became a multifaceted concept with inconsistent definitions of social support. To summarize the typical definitions of social support (as shown in Table 1), Perkins (12), Cohen (10), Smedley et al. (11) and other relevant domestic and international researchers have interpreted and analyzed the concept of social support from different perspectives and research purposes.

**Table 1:** Conceptualization of social support.

**Scholars**	**Definitions of social support**
Caplan et al. (9)	Social support is a continuous social cohesion in which individuals interact with other people and social networks as a support system to meet individual needs or enhance their adaptability
Cohen et al. (10)	Social support is the resources provided by others to help a person cope with the stresses
Smedley et al. (11)	Social support can be divided into tangible and intangible social support. For example, when children participate in competitive competitions, the economic support provided by family members is tangible social support, while the competitive suggestions provided by teachers and classmates are invisible social support

As social support research has become more advanced, its classification has been characterized by a diversity of approaches according to the respective research needs. (13) divided social support into four dimensions: belongingness, satisfying self-esteem, favorability, and materiality (14). (15) classified social hosting into 6 categories based on the functions: emotional, network, informational, material, instrumental and nurturing support. (16) categorized social support in 6 areas: material help, behavioral assistance, intimate behaviors, guidance, feedback, and positive behaviors. The social support questionnaire by Matt G. M. vanderpur categorized the 11 support behaviors into 3 types: moral support, practical support, and social interaction.

Current scholars classify social support according to the different subjects of support, which means that social support is divided into formal social support and informal social support (17). Social support has always followed two parallel lines, one is the altruistic support that exists in the altruism of the lower classes informal support; and the other is the utilitarianism of the upper classes toward the lower classes, i.e., formal support (18). In this paper, formal social support is social support provided by the government or social organizations for disadvantaged groups. This type of social support subject has specific functions and formal organizational norms, clear standards, and procedures. Informal social support is moral or material help provided by individuals or groups who do not have specific functions or formal norms of social support, and either not seek any financial reward for their social behaviors. Informal subjects of social support include individuals, families, neighbors, colleagues, friends, etc.

### Healthcare Services

Yali et al. pointed out that the three main demand for healthcare services for older people: healthcare services, health guidance, and self-care knowledge (19). Patients with disease need information on management (both conventional and non-conventional), relevant targeted advice and access to appropriately skilled professionals should be viable components of healthcare services (20). The American Geriatrics Society defines continuity of care as a series of healthcare activities designed to ensure coordination and continuity of healthcare services for patients as they move between care sites, to prevent or reduce deterioration in the health status of high-risk patients. Studies show that <20% of ill people seek care at a health facility, 10% visit a traditional healer and more than 50% engage in self-care at home in Burkina Faso. The main reason for not seeking professional care is “not having enough money” (21). Gnawali et al. (20) defined healthcare services utilization as outpatient visits and inpatient care, outpatient visits mean the total number of visits by sick households to a health facility during the recall period, inpatient care means any hospitalization for at least 1 day during 1 month recall period. Most scholars define healthcare services as therapeutic health nursing service, and some scholars emphasize the importance of preventive health nursing service.

### Influencing Factors of Social Support

Several studies have focused on the impact of social support on the health status, happiness, and quality of life of older people. Social support has become one of the most important factors influencing the demand for healthcare services for older people, and studies have shown that social support has a significant impact on the types, number and cost of healthcare services utilized by older people. Due to the hierarchical property, they can be divided into curative and preventive healthcare services, with curative healthcare services being divided into outpatient and inpatient areas. Thus, the impact of social support on the different levels of healthcare utilization should be differentiated, with most studies focusing on the factors influencing healthcare utilization (22) and very limited research on the core variables of social support.

In China, the persistence of the urban-rural dichotomy has led to significant differences in healthcare services for older people in urban and rural areas. The level of health care services for older people in urban China is generally higher than that in rural areas. Urban and rural older people also differ in terms of the factors that influence the use of healthcare services, and rural older people are more dependent on the social and child support. In urban areas, the family gives more material support, while in rural areas, spiritual support is the most important aspect of family support. Studies have shown that gender, household registration, pre-retirement occupation, education background, health status of yourself and your spouse, self-care ability, household income, and labor migration all have a significant impact on healthcare utilization of older people.

## Research Framework

Most scholars have focused on the impact of social support on the utilization of healthcare services for older people, focusing on specific groups of older people, such as those living alone and the disabled (23), but lacking a comprehensive approach to the impact of social support on the utilization of healthcare services for older people in different living environments, from both urban and rural perspectives (24).

The existing research on the connotation of healthcare utilization among older people is focused on curative healthcare services, but there is a lack of research on preventive healthcare services, which is of great importance in China. Therefore, this paper will explore the connotation of health care services for older people and expand it to include preventive healthcare services in the analysis framework, and explore the impact of social support on the use of preventive healthcare services for older people (25).

In the selection of social support variables, most scholars only consider the availability of health insurance and old-age insurance among formal support, but rarely involve the issue of satisfaction with health insurance and old-age insurance, while with the general increase in the level of formal support, the more important issue is the quality of health insurance and old-age insurance (26). Therefore, satisfaction with health insurance and old-age insurance should be included in the model when examining the extent to which formal social support affects the use of healthcare services by older people (27). Among informal social support, more scholars have considered the influence of intergenerational support (28), or child support, on older people's healthcare utilization, although a few scholars have begun to focus on the influence of social relationships such as marital relationships (husband and wife), community relationships and friendships on older people's healthcare utilization (29–31). It is important to include the quality of neighborhood relationships in the model, as they are an important part of the social relationships of older people in their daily lives (32).

**Figure 1:**
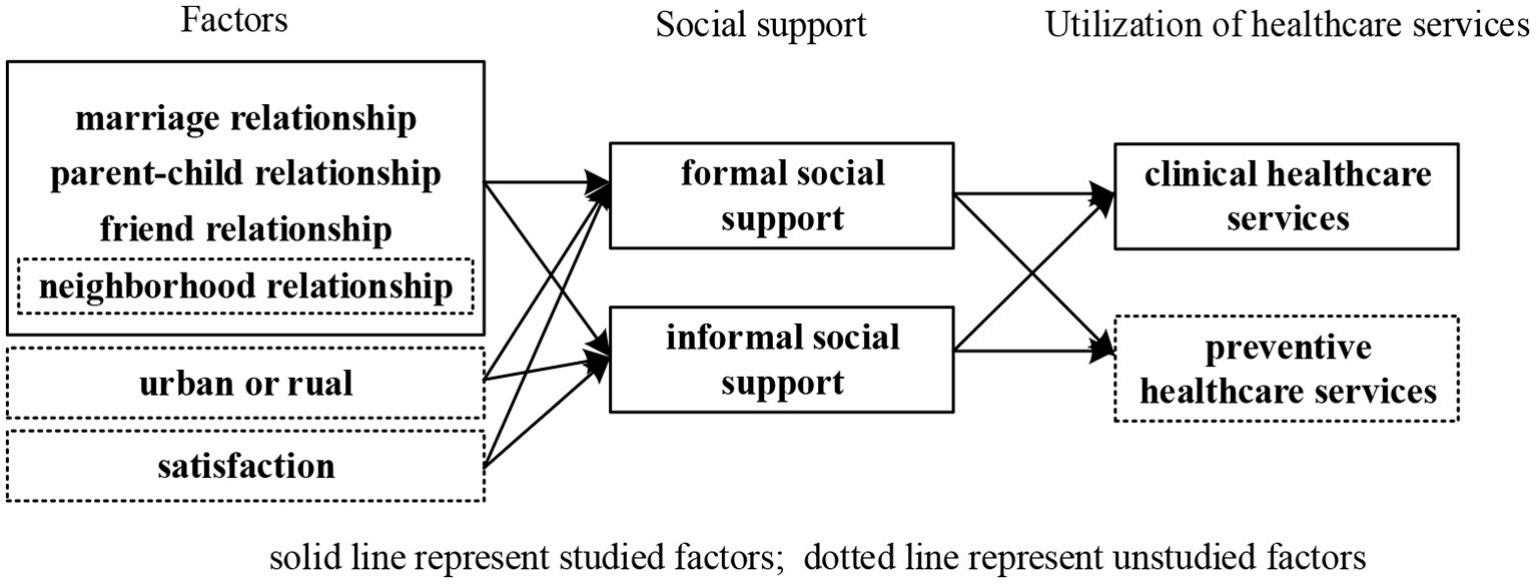
Current status of social support research.

This paper attempts to explore the impact of social support on healthcare utilizations among older people in both urban and rural areas (Figure 1).

The Andersen model (1995) is an explanatory framework for the analysis the utilization of healthcare, which divides the utilization of healthcare influencing factors into three broad categories: dispositional characteristics, enabling factors and demand factors. Previous studies have found that propensity factors such as age, gender, education, and health knowledge have a significant impact on healthcare utilization, while demand factors such as health status and type of chronic disease have a significant impact on healthcare utilization. In US, people with lower income are less likely to seek care (33), the same “pro-rich” health inequalities exist in China (34). The health insurance system has increased access to healthcare for older people in China, reducing health inequalities and reducing the burden of healthcare on older people (35).

**Figure 2:**
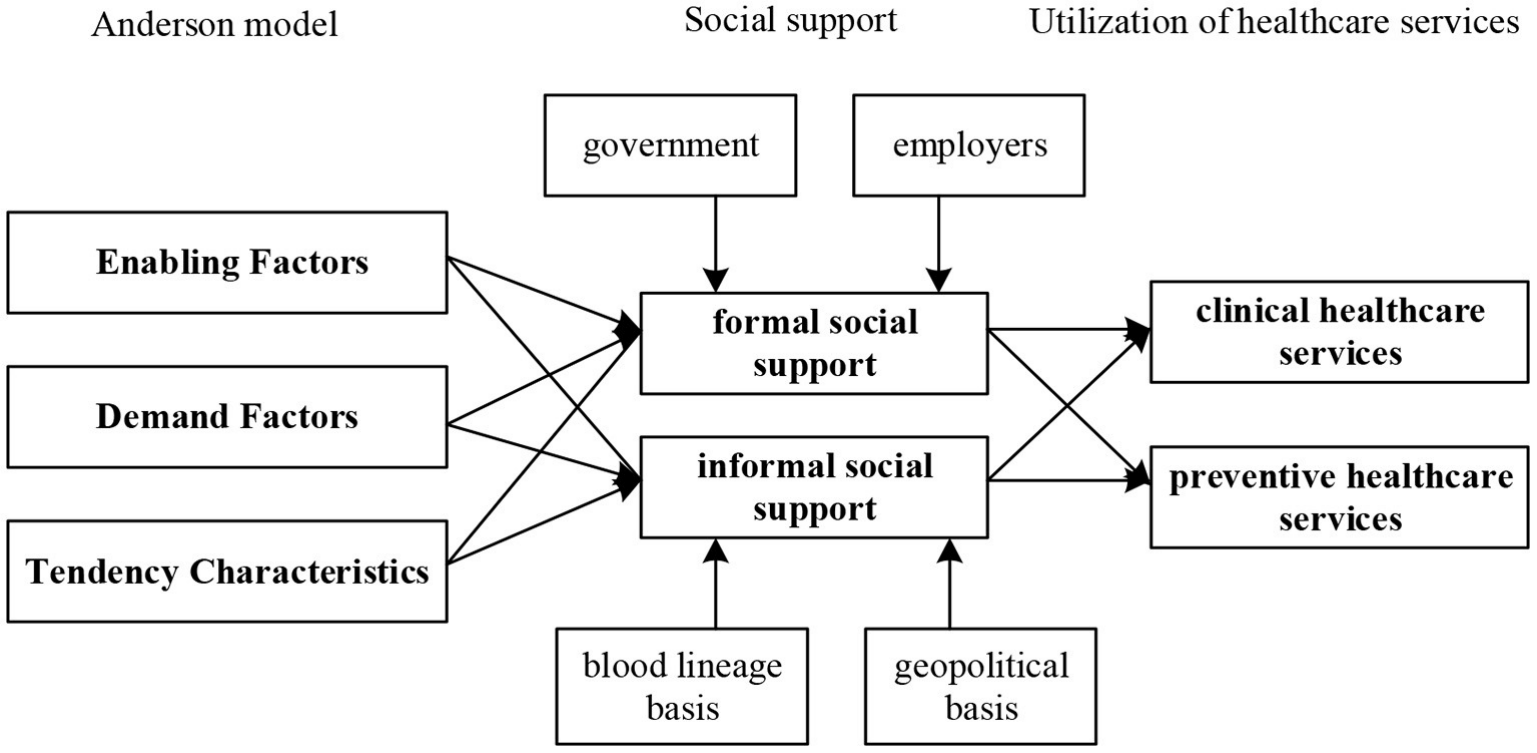
Research framework.

Therefore, we divide the influencing factors of social support into enabling factors, dispositional factors, and demand factors according to the Anderson model. This paper will study the influence of formal and informal social support on therapeutic and preventive healthcare services, respectively (Figure 2).

The advantage of this study lies in the inclusion of formal and informal support into the influencing factors of the utilization of healthcare services for the older people, as well as the inclusion of preventive healthcare services, which are rarely considered in healthcare services, into the analysis of the paper, and the differentiation of influencing factors between urban and rural areas.

## Methods and Data

### Data Sources and Methods

Shaanxi is located in the core of China's geographical location, and its economic and social development level and aging degree are at a medium level in the whole of China, so it is of great significance to choose Shaanxi province. According to the regional distribution characteristics of shaanxi province, field survey with stratified random sampling method, choice of Xi'an, Baoji, Han zhong city as the first layer, each city randomly select 2 counties as the second layer, a total of 6 counties, then randomly select 2 township from each county the third layer, finally in each township randomly selected two community as the fourth floor, A total of 24 communities are surveyed. The survey is conducted among people over the age of 60. Before data collection, we orally introduce the background, content and purpose of the study to the respondents, and only when the respondents confirm their willingness to participate in the survey, our investigators will start the survey. All procedures performed in this study are ethical. All questionnaires are completed anonymously with the verbal informed consent of each participant.

The research was endorsed by the Department of Social Sciences of the Ministry of Education and the Ethics Committee of Xi'an Jiaotong University, approval number is 2018–1200. Because no personal privacy was involved, the survey was completed anonymously and older people who participated in the survey waived signing the informed consent form. A total of 980 questionnaires were distributed in this survey, with 948 valid questionnaires and a valid return rate of 6.7%. From this study, 683 questionnaires related to the research topic were selected for analysis.


(1) $$\begin{array}{r} n=\frac{t^{2}\sigma^{2}}{err^{2}} \end{array}$$

In the sign 1, t is the sample standard deviation, err is the error, 95% confidence and 3% margin error, a minimum of 544 samples are required. Six hundred eighty-three Respondents is eligible.

Since (36) studied the problems such as the upper limit, lower limit or extreme value of explained variables, this kind of research has attracted extensive attention of scholars. In order to commemorate Tobin's contribution to such models, the models with limited values of explained variables and selection behaviors are called Tobit model. This kind of model actually contains two kinds of equations, one is the discrete data model reflecting the selection problem; One is a constrained continuous variable model (37). The second model tends to be the more interesting part of the literature. Tobit model which follows the concept of large likelihood method is a suitable choice to analyze the influencing factors. Logit model generally adopts maximum likelihood estimation method for parameter estimation (38). It is not appropriate to use linear regression model for the variables to be studied in this paper, so Tobit model and Logit model are needed.

Using the Anderson model as the basic explanatory framework, this paper uses the Tobit model to analyses the impact of social support for older people on the utilization of therapeutic healthcare services, and the Logit model to analyze the impact of living arrangements and social support on the utilization of preventive healthcare services for older people, and finally proposes recommendations to help older people make better use of healthcare services from the perspective of social support.

### Variables Selection and Descriptive Statistics

Most scholars choose variables such as number of doctor visits and days in hospital over a certain period to measure the level of healthcare utilization, so this paper selected the number of outpatient visits and days in hospital to measure the utilization of therapeutic healthcare services. The number of visits is the number of times older people visited a health facility in the past month. The number of inpatient days refers to the cumulative number of days spent in hospital at all levels of care in the past year. Table 2 shows the description and multicollinearity test of the selected independent variables in this paper. VIF values are all <10. Therefore, the variables selected in this paper do not have multicollinearity.

**Table 2:** Influencing factors of mental health of older people.

**Categorical variables**	**Name**	**Meaning**	**VIF**
Healthcare service utilization	Outpatient	Distance variable	2.014
	Hospitalization	Distance variable	1443
	Health checkup	1 = Have,0 = No	1.665
Formal social support	Health insurance	1 = Have,0 = No	2.438
	Pension	1 = Have,0 = No	2.315
	Frequency of contact with government staff	1 = High, 0 = Low	2.571
	Health insurance satisfaction	1 = Satisfied, 0 = Dissatisfied	3.645
	Pension insurance satisfaction	1 = Satisfied, 0 = Dissatisfied	2.415
Informal social support	Way of living	1 = Livealone, 0 = Not living alone	1.262
	Children's communication frequency	1 = High, 0 = Low	1.741
	Children's financial support	1 = High, 0 = Low	1.542
	Frequency of helping each other with neighbors	1 = High, 0 = Low	1.385
	Frequency of communication with neighbors	1 = High, 0 = Low	2.347

Activities of daily living (ADL) include the following indicators :“Whether you can participate in outdoor activities,” “whether you can walk up and down stairs,” “whether you can take a bath independently,” “whether you can get on and off bed by yourself,” “whether you can wash your face and comb your hair independently,” “Whether you can dress independently,” “whether you can go to the bathroom independently, “whether you can eat independently.”

Among the surveyed older people, the youngest is 60 years old and the oldest is 92 years old, with an average age of 70.41 years old; the ratio of men to women is about 0.6:1, with more female older people than male older people; in terms of education level, the percentage of older people with elementary school education and below reached 43.48%, while those with college education and above only accounted for about 8%, and the overall education level of the surveyed older people is low; in terms of household registration, the ratio between urban and rural households is 1.3:1, with slightly more older people living in urban households than in rural households; in terms of self-care ability, the mean value of Activities of daily living (ADL) is 0.05, meaning that the majority of older people are not physically impaired; in terms of living alone, the ratio between older people living alone and those not living alone is about 0.2:1, with most of the surveyed older people living with their children and other family members; in terms of marriage type, the largest number of older people are married, accounting for 71.5%. In terms of marriage type, the largest number of older people are married, accounting for 71.60%, followed by widowed, accounting for 25.48%. Descriptive statistics are shown in Table 3.

**Table 3:** Descriptive statistics.

**Variables**	**Classification**	**Number**	**Frequency (%)**
Age	60 years old and above	Mean value.70.41	
		Standard deviation.7.08	
Gender	Male	265	38.80
	Female	418	61.20
Education	Elementary school and below	297	43.48
	Junior high school	193	28.26
	High school/junior high school	138	20.21
	College	38	5.56
	Bachelor's degree or above	17	2.49
Marriage	Unmarried	10	1.46
	Married	489	71.60
	Divorced	10	1.46
	Widowed	174	25.48
Household registration	Urban	388	56.81
	Rural	295	43.19
Whether living alone	No	557	81.55
	Yes	126	18.45
Activities of daily living (ADL)	0~6	Mean value.0.05	
		Standard deviation.0.43	

Table 4 shows the empirical results of the impact of formal and informal social support on utilization of outpatient healthcare service. Table 5 presents the empirical results of the impact of formal and informal social support on the utilization of inpatient healthcare services. Model 1 only considers the influence of control variables, and the results show that age and ADL have significant influence on the number of outpatient visits. Model 2 considers the influence of both formal social support and informal social support on the utilization of therapeutic healthcare services based on model 1, and the results show that among the control variables, age, marriage, income, and ADL significantly influence the number of outpatient visits for older people, and the results showed that age, marriage, income and ADL significantly influenced the number of outpatient visits, while health insurance, frequency of contact with government staff, health insurance satisfaction and pension insurance satisfaction positively influenced the number of outpatient visits. In formal social support, health insurance, frequency of contact with government staff, health insurance satisfaction, and pension insurance satisfaction positively affected the number of outpatient visits. For informal social support, children's communication frequency and financial support, frequency of helping each other with neighbors, frequency of communication with neighbors had significant positive effect on the number of outpatient visits.

**Table 4:** The impact of formal and informal social support on utilization of outpatient healthcare service.

**Variable**	**Model 1β (*t*)**	**Model 2 (*t*)**	**Model 3 (*t*) rural**	**Model 4 (*t*) urban**
**Control variable**
Gender	0.715 (1.353)	0.788 (1.641)	0.742 (1.556)	0.972 (2.014)
Age	0.748^**^ (−1.625)	0.798^**^ (−1.353)	0.568^**^ (−1.412)	1.101^**^ (−3. 068)
Marriage	0.688 (1.389)	0.728^**^ (1.471)	1.209 (2.487)	1.716 (4.241)
Education	0.226 (0.376)	0.247 (0.515)	1.728 (4.018)	1.435 (3.1841)
Political status	0.128 (0.291)	0.154 (0.276)	0.062 (0.183)	0.096 (0.217)
Income	0.084 (0.188)	0.168^***^ (0.343)	2.352^***^ (6.132)	1.352* (2.468)
ADL	−0.099^***^ (−0.321)	−0.309^**^ (−0.842)	−0.472^**^ (−1.021)	−0.844^**^ (−1.645)
**Formal social support**
Health insurance		0.912* (2.154)	1.032^***^ (2.388)	1.654 (3.246)
Pension		0.819 (1.808)	0.916^**^ (2.016)	0.814 (1.983)
Frequency of contact with government staff		1.35 (7.827)	3.992 (7.827)	1.471 (2.996)
Health insurance satisfaction		1.009^***^ (2.038)	1.797* (3.466)	1.531^***^ (2.774)
Pension insurance satisfaction		1.494^***^ (2.816)	2.014 (3.792)	3.083^***^ (7.827)
**Informal social support**
Children's communication frequency		2.037^**^ (5.187)	3.169^***^ (8.452)	1.488* (3.291)
Children's financial support		2.154^**^ (5.663)	2.037^**^ (5.316)	1.037 (2.435)
Frequency of helping each other with neighbors		1.814^***^ (4.021)	0.947 (1.769)	1.382^***^ (2.971)
Frequency of communication with neighbors		1.114^**^ (1.984)	1.105 (2.019)	1.392^**^ (3.128)
*P*-Value	0.000	0.000	0.000	0.000

***, **, and * were significant at 1, 5, and 10% levels respectively. t-values are in parentheses.

**Table 5:** The impact of formal and informal social support on the utilization of inpatient healthcare services.

**Variable**	**Model 5 (*t*)**	**Model 6 (*t*)**	**Model 7 (*t*) rural**	**Model 8 (*t*) urban**
**Control variable**
Gender	0.642 (1.304)	0.712 (1.681)	0.891 (1.922)	0.836 (1.748)
Age	1.613^**^ (2.682)	1.743^***^ (4.011)	1.568^***^ (3.354)	2.841^***^ (5.991)
Marriage	0.841 (1.781)	0.992 (1.994)	1.442 (2,762)	1.543 (3,475)
Education	0.887 (1.876)	0.976^**^ (1.623)	0.774^**^ (1.669)	0.697 (1.439)
Political status	0.066 (0.182)	0.078 (0.114)	0.091 (0.253)	0.079 (0.174)
Income	1.671^**^ (3.871)	1.439^**^ (2.954)	1.441^**^ (2.878)	1.935 (4.397)
ADL	−1.899^***^ (−3.624)	−2.309^***^ (3.471)	−2.472^***^ (4.425)	−2.844^**^ (−4.893)
**Formal social support**
Health insurance		0.712^**^ (1.367)	0.647^***^ (1.291)	1.382 (3.446)
Pension		0.819^**^ (1.445)	0.916^***^ (2.113)	0.814 (1.509)
Frequency of contact with government staff		2.357 (3.861)	4.114 (5.911)	2.361 (3.348)
Health insurance satisfaction		1.887^**^ (3.618)	1.231^*^ (3.772)	2.006^***^ (4.045)
Pension insurance satisfaction		1.466^**^ (2.433)	1.096 (1.984)	2.761^**^ (3.998)
**Informal social support**
Children's communication frequency		1.382^*^ (2.435)	3.241^***^ (3.967)	2.336^**^ (3.892)
Children's financial support		2.438 (4.951)	2.589^**^ (4.769)	1.613 (2.315)
Frequency of helping each other with neighbors		1.471^***^ (2.492)	0.928 (1.661)	1.893^***^ (3.945)
Frequency of communication with neighbors		1.997^***^ (3.774)	1.308 (2.953)	2.412^**^ (4.631)
*P*-Value	0.000	0.000	0.000	0.000

***, **, and * were significant at 1, 5, and 10% levels respectively. t-values are in parentheses.

Model 5 only considered the effect of control variables on hospitalization of older people, age, income, and ADL had a significant effect on the number of days older people spent in hospital. Model 6 considered both formal and informal social support on the use of therapeutic healthcare services based on model 5. In the formal social support, Health insurance, Pension, Health insurance satisfaction, and Pension insurance satisfaction significantly and positively influenced the number of days of hospitalization of older people. In informal social support, frequency of children's communication, helping each other with neighbors, communication with neighbors significantly and positively influenced the number of days in hospital. The number of days in hospital was positively affected by Children's communication frequency, Frequency of helping each other with neighbors and Frequency of communication with neighbors. In summary, social support significantly influenced the use of curative healthcare services by older people.

Model 3 and Model 7 show the effect of formal and informal social support on the use of therapeutic healthcare services for rural older people. The results show that the control variables age, income, and ADL have significant effect on the number of outpatient visits and the number of days in hospital for rural older people, while only education has a significant effect on the number of days in hospital. Among the formal social support variables, health insurance, pension, frequency of contact with government staff and health insurance satisfaction had significant positive effect on the number of outpatient visits and days in hospital for rural older people. Among the informal social support, children's communication frequency and financial support had significant positive effect on the number of outpatient visits and days in hospital for the rural older people. Thus, social support significantly influenced the use of therapeutic healthcare services by rural older people.

Models 4 and 8 show the effect of formal and informal social support on the utilization of therapeutic healthcare services by urban older people. The results showed that the control variables age, income, and ADL had significant effect on the number of outpatient visits for urban older people, age and ADL had significant effect on the number of days in hospital for urban older people. Among the formal social support variables, health insurance satisfaction and pension insurance satisfaction had significant positive effect on the number of outpatient visits and days in hospital for the urban older people. For informal social support, children's communication frequency, frequency of helping each other with neighbors and communication with neighbors had significant effect on the number of outpatient visits and days in hospital for urban older people. In conclusion, social support significantly influenced the use of therapeutic healthcare services by urban older people.

### Logistic Regression Analysis of Preventive Healthcare Services

We used binary logistic regression analysis for Preventive Healthcare services. Table 6 presents the results of the empirical tests of the effect of formal and informal social support on the utilization of preventive healthcare services. Model 1a considers the effect of control variables only and the results show that Age, Education and ADL significantly affect the utilization of preventive healthcare services for older people, and Age and ADL are highly significant in all four models. Model 2 considered both formal and informal social support on preventive healthcare utilization based on model 1a, and the results showed that Age, Education, and Income significantly and positively influenced preventive healthcare utilization, while ADL significantly and negatively influenced preventive healthcare utilization. The higher the level of education (OR = 2.369), income (OR = 3.331) and age (OR = 1.398), the more likely the older adult was to use preventive healthcare services; the better the ability to care for oneself, the less likely the older adult was to use preventive healthcare services (OR = 0.714). Among the formal social supports, Frequency of contact with government staff, Health insurance satisfaction, and Pension insurance satisfaction significantly and positively influenced the use of preventive healthcare services. That is, the more frequent the contact with government staff (OR = 3.209), the higher the satisfaction with health insurance (OR = 1.384), and the higher the satisfaction with pension insurance (OR = 1.701), the more likely it was that older people would utilize preventive healthcare services. For informal social support, Children's communication frequency, Children's financial support, Frequency of helping each other with neighbors, Frequency of communication with neighbors all positively influenced older people's use of preventive healthcare services. That is, the more frequently older people communicated with their children (OR = 2.371), the greater the financial support provided by their children (OR = 3.214), the more frequently they helped each other with neighbors (OR = 3.498), and the more frequently they communicated with neighbors (OR = 2.359), the more likely older people were to use preventive healthcare services. In conclusion, social support significantly influenced the use of preventive healthcare services by older people.

**Table 6:** The impact of social support on utilization of preventive healthcare services.

**Variable**	**Model 1a (OR)**	**Model 2b (OR)**	**Model 3c (OR) rural**	**Model 4d (OR) urban**
**Control variable**
Gender	0.032 (1.004)	0.056 (1.372)	0.047 (1.483)	0.081 (1.441)
Age	0.687^***^ (1.433)	0.676^**^ (1.398)	0.898^**^ (1.254)	0.976^**^ (1.677)
Marriage	0.090 (1.231)	0.082 (1.967)	0.076 (1.887)	0.092 (1.844)
Education	0.254^**^ (2.387)	0.372^**^ (2.369)	0.364^***^ (2.941)	0.497 (2.772)
Political status	0.093 (1.693)	0.088 (1.892)	0.076 (1.976)	0.071 (1.856)
Income	1.274 (3.532)	1.399 (3.468)^*^	1.382 (2.764)	1.765 (2.879)^**^
ADL	−0.438^***^ (0.417)	−0.502^**^ (0.714)	−0.665^***^ (0.810)	−0.492^***^ (0.788)
**Formal social support**
Health insurance		0.472^*^ (2.362)	0.414 (3.891)	0.569 (4.493)^**^
Pension		0.878 (2.141)	0.927 (1.898)	0.946 (2.984)^**^
Frequency of contact with government staff		1.448^**^ (3.209)	1.392^***^ (3.192)	1.628 (2.376)
Health insurance satisfaction		0.778^**^ (1.384)	0.982^*^ (1.887)	0.951^**^ (1.662)
Pension insurance satisfaction		0.892^**^ (1.701)	0.483 (1.983)	0.986^**^ (1.462)
**Informal social support**
Children's communication frequency		0.924^**^ (2.371)	0.887^***^ (2.549)	0.941^**^ (2.203)
Children's financial support		0.813^*^ (3.214)	0.874^**^ (3.926)	0.762 (3.835)
Frequency of helping each other with neighbors		1.994^**^ (3.498)	1.057 (2.247)	1.148^**^ (3.997)
Frequency of communication with neighbors		1.541^**^ (2.359)	1.167 (2.454)	1.909^***^ (2.328)
*P*-Value	0.000	0.000	0.000	0.000

***, **, and * were significant at 1, 5 and 10% levels respectively. OR values are in parentheses.

Model 3c indicates the effect of formal and informal social support on the use of preventive healthcare services for rural older people. The results show that among the control variables, Age, Education, and ADL significantly affect the use of preventive healthcare services for rural older people. Among the formal social support variables, Frequency of contact with government staff, Health insurance satisfaction, and Pension insurance satisfaction significantly and positively influenced the utilization of preventive healthcare services by rural older people. That is, the more frequent the contact with government staff (OR = 3.192), the higher the level of health insurance (OR = 3.891), and the higher the level of pension insurance (OR = 1.898), the more likely older people were to use preventive healthcare services. Among the informal social supports, Children's communication frequency, Children's financial support, and Frequency of helping each other with neighbors significantly and positively influenced the use of preventive healthcare services by rural older people. The more frequently older people communicated with their children (OR = 3.549) and the more financial support their children provided (OR = 2.926), the more likely older people were to utilize preventive healthcare services. In summary, social support significantly influenced the use of preventive healthcare services by rural older people.

Model 4d, which refers to the effect of formal and informal social support on the use of preventive healthcare services by urban older people, shows that among the control variables, Age, Income, and ADL significantly affect the use of preventive healthcare services by urban older people. Among the formal social support variables, Frequency of contact with government staff, Health insurance satisfaction, and Pension insurance satisfaction significantly and positively influenced the utilization of preventive healthcare services among the urban older people. That is, the more frequent the contact with government staff (OR = 2.376), the higher the satisfaction with health insurance (OR = 1.662), and the higher the satisfaction with pension insurance (OR = 1.462), the more likely older people were to use preventive healthcare services. In terms of informal social support, Frequency of helping each other with neighbors and Frequency of communication with neighbors significantly and positively influenced the use of preventive healthcare services by urban older people. That is, the more frequently older people helped each other with neighbors (OR = 3.997) and the more frequently older people communicated with neighbors (OR = 2.328), the more likely they were to use preventive healthcare services. In conclusion, social support significantly influenced the use of preventive healthcare services by older urban residents.

### Oaxaca Blinder Decomposition of Urban-Rural Healthcare Service Utilization Differential Factors

In the empirical analysis, the outpatient, inpatient and preventive healthcare services above are combined into the actual healthcare services used by older people. To further decompose the contribution of different factors in the variability of healthcare service utilization among older people, this paper performs an Oaxaca Blinder decomposition of healthcare service utilization among older people in urban and rural areas based on the model above. Table 7 shows the results for Oaxaca Blinder Decomposition.

**Table 7:** Oaxaca blinder decomposition.

**Control variable**	**Regression coefficient**	**Robust standard errors**	**Percentage (%)**
Gender	−0.057	0.007	−3.121
Age	0.014	0.002	4.762
Marriage	0.004	0.006	0.893
Education	0.012	0.004	6.961
Political status	0.007	0.003	8.927
Income	−0.114	0.063	−6.302
ADL	−0.067	0.067	−14.396
**Formal social support**			−2.276
Health insurance	0.103	0.094	5.125
Pension	−0.081	0.815	−5.882
Frequency of contact with government staff	0.074	0.072	8.363
Health insurance satisfaction	0.152	0.714	18.182
Pension insurance satisfaction	0.022	0.091	26.051
**Informal social support**			51.839
Children's communication frequency	0.086	0.019	6.321
Children's financial support	0.147	0.087	16.053
Frequency of helping each other with neighbors	0.176	0.098	18.014
Frequency of communication with neighbors	0.018	0.104	10.049
Feature effect	0.012	0.147	70.3
Coefficient effect	0.452	0.382	29.7
Urban	0.394	0.471	10.8
Rural	0.281	0.265	18.9

From the previous section, the difference in healthcare service utilization between urban and rural older people can be decomposed into two parts: one part of the difference is caused by the difference between urban and rural residents on variables such as antecedent factors, enabling factors, demand factors and policy factors, called the characteristic effect, which explains about 70.3% of the difference; the other part of the difference is the coefficient effect, i.e., if urban and rural older people are identical on all variables, the difference is only due to these variables (regression coefficients before the variables). The coefficient effect is divided into two parts: the coefficient “premium” of urban older people relative to urban and rural older people as a whole, which accounts for 10.8% of the total difference; and the “premium” of urban and rural older people as a whole to rural older people, which accounts for 18.9% of the total difference. The coefficient effect accounts for 29.7 per cent of the total difference, and the paper suggests that this can be explained by unobserved differences in healthcare habits and attitudes between urban and rural older people.

## Discussion

Both formal and informal social support had a significant impact on outpatient, inpatient, and preventive healthcare services for older people. In terms of age characteristics, older people tend to use healthcare services more; the higher the level of ADL, the lower the demand for healthcare service use and the actual use, with a negative effect of ADL on healthcare service use, this is consistent with the conclusion of SA Snih's study (39). Education level has no effect on the use of outpatient healthcare services, but has a significant effect on inpatient and preventive healthcare services for rural older people, whereas it has no significant effect on healthcare services for urban older people. This suggests that there is a tendency for healthcare services to be pro-intellectualized, given the generally low level of education in rural China. In rural areas, people with a good level of education have a more correct understanding of inpatient care, as well as preventive healthcare services, and can promote their use of these services (40).

Firstly, there are significant urban-rural differences in the impact of formal support on the utilization of healthcare services for older people. Rural older people place greater importance on the quantity of social support. This is consistent with the conclusion of B Pan's study, but his study did not consider the influence factors of the formal support of the urban older people on healthcare services (41), while urban older people place greater emphasis on the quality of social support. Specifically, the pensions and health insurance had a significant impact on the use of therapeutic healthcare services among rural older people, and the satisfaction with pensions and health insurance had a significant impact on therapeutic healthcare services among urban older people, reflecting the imbalance between urban and rural social security. Although the provision of free preventive healthcare services in rural areas has largely compensated for the lack of benefits for rural older people, the effectiveness of this policy depends on primary public officials. In contrast, urban older people mostly pay for their own medical check-ups, so the level of pensions and health insurance has a significant impact on preventive healthcare services (42).

Secondly, A's study describes the importance of traditional culture and children's support to older people in China, but he does not distinguish the huge older people group in China (43). This paper found that the concept of raising children for old age is evident in the utilization of healthcare services by rural older people, whereas the concept that close neighbors are better than distant relatives have a significant impact on the utilization of healthcare services by urban older people. In terms of informal social support, both moral and financial support from children have a significant impact on the use of therapeutic and preventive healthcare services for rural older people, showing that rural older people are more likely to be influenced by their children in their daily lives, as they do not have sufficient income and need financial support from their children for healthcare services, and that the phenomenon of “raising children for old age” still exists in rural areas. Due to the one-child policy, most urban older people have only one child and their children are not nearby, so urban older people rely more on neighborhood support, while child communication has a significant impact on the use of preventive healthcare services among rural older people, suggesting that older people still consult their children most of the time, which is consistent with the research conclusion of Zhang et al. (44).

## Conclusions

Using research data from Shaanxi Province in 2019, this paper examines the impact of formal and informal support on outpatient, inpatient, and preventive healthcare services for older people, provides an in-depth analysis of the differences in the impact of social support on healthcare services utilization between urban and rural older people and analyses the contribution of various factors to the impact. The study finds that rural older people place greater importance on the quantity of formal social support, while urban older people place greater importance on the quality of formal social support. The phenomenon of raising children for old age was evident in the use of healthcare services by rural older people, while the phenomenon of distant relatives being better than close neighbors was evident in the use of healthcare services by urban older people. Free preventive healthcare services in rural China have largely compensated for the lack of health benefits for rural older people. We suggest that the government should introduce policies to guarantee healthcare services for the older people in rural areas as part of the national development strategy. Families should be given more responsibility and traditional culture should be maintained by providing support for older people. Rural older people should get more free health care services.

## Data Availability Statement

The raw data supporting the conclusions of this article will be made available by the authors, without undue reservation.

## Ethics Statement

This study is approved by the Department of Social Sciences of the Ministry of Education, PRC and the Medical Ethics Committee of Health Science Center of Xi'an Jiaotong University (Approval Number 2018-1200). Written informed consent for participation was not required for this study in accordance with the national legislation and the institutional requirements.

## Author Contributions

ZC and HH completed the analyzing, writing, and modification of the manuscript. HH examined and supervised the study. All authors have read and agreed to the published version of the manuscript.

## Funding

This study was funded by the Major Projects of Philosophy and Social Science Research of the Ministry of Education (18JZD045), China Postdoctoral Science Foundation (2019M663773), and Basic research funds of central universities (SK2021009).

## Conflict of Interest

The authors declare that the research was conducted in the absence of any commercial or financial relationships that could be construed as a potential conflict of interest.

## Publisher's Note

All claims expressed in this article are solely those of the authors and do not necessarily represent those of their affiliated organizations, or those of the publisher, the editors and the reviewers. Any product that may be evaluated in this article, or claim that may be made by its manufacturer, is not guaranteed or endorsed by the publisher.
